# Gene Expression Differences in Muscle and Adipose Tissue Help Explain Variation in Meat Tenderness Across USDA Carcass Grades in Beef and Fat Classes in Sheep †

**DOI:** 10.3390/ani16162493

**Published:** 2026-08-11

**Authors:** Madison Schumacher, Jane Ann Boles, Jennifer M. Thomson

**Affiliations:** Department of Animal and Range Sciences, Montana State University, Bozeman, MT 59717, USA; madison.schumacher15@gmail.com (M.S.); jboles@montana.edu (J.A.B.)

**Keywords:** marbling, USDA quality grade, RNA-seq, differential gene expression, longissimus muscle, adipose tissue, meat tenderness, shear force, lipid metabolism, beef cattle and sheep

## Abstract

Meat tenderness is a critical quality trait that varies even among carcasses of similar USDA quality grades. In this comparative study of beef cattle and sheep, we investigated how gene expression profiles in muscle and adipose tissues are associated with carcass quality grades and Fat Classes. Fifteen steers and fifteen wethers were raised to produce carcasses spanning Standard, Select, and Choice USDA quality grades (beef) or varying Fat Classes (sheep). In beef, higher quality grade was associated with significantly improved tenderness (lower shear force), while sheep tenderness did not differ consistently across Fat Classes. Exploratory RNA sequencing identified hundreds to thousands of differentially expressed genes (DEGs) between high- and low-quality or fatness groups, particularly in sheep muscle. DEGs were enriched in pathways related to muscle growth, lipid metabolism, and immune signaling. While these transcriptomic differences suggest biological processes associated with carcass traits, the findings are correlational, should be interpreted as hypothesis-generating, and require further validation in larger independent populations. These exploratory findings suggest species-specific differences in the relationship between fatness and tenderness and identify candidate molecular pathways for future investigation.

## Simple Summary

Meat tenderness is one of the main reasons people like (or dislike) a steak or chop, but it can vary a lot even when carcasses receive the same quality grade. In this study, we compared beef cattle and sheep raised to produce carcasses with different levels of fatness and quality. We measured carcass traits and used a standard tenderness test after cooking. In cattle, higher-quality grade beef was more tender, whereas lamb tenderness did not change clearly across fatness groups in this trial. We also analyzed gene activity in muscle and fat using RNA sequencing, which shows which genes are “turned on” or “off” in a tissue. Animals with higher fatness or higher quality grade showed many differences in genes related to muscle growth, energy use, fat formation and storage, and immune system responses. These results suggest that differences in fat deposition characteristics that are linked to quality grades are associated with measurable biological changes in both muscle and fat and identify candidate molecular pathways that may help guide future studies aimed at improving the consistency of tenderness for consumers.

## 1. Introduction

Meat tenderness is widely recognized as one of the most important quality attributes influencing consumer satisfaction, repeat purchases, and willingness to pay for beef and lamb products [1]. Marbling, or intramuscular fat content, contributes to juiciness and flavor and can improve tenderness; however, it remains an imperfect predictor of eating quality. Even within the same USDA quality grade, substantial variation in tenderness has been documented. For example, Rodriguez et al. [2] reported a weak correlation (r ≈ −0.13) between marbling score and shear force in Brangus beef. Similarly, Vierck et al. [3] found that marbling texture had minimal effects on beef palatability and did not influence Warner–Bratzler shear force across carcasses representing multiple quality grades. Together, these findings underscore the limitations of relying solely on USDA quality grades to predict tenderness and highlight the need for a deeper understanding of the biological mechanisms underlying meat quality.

High-throughput transcriptomic approaches, such as RNA sequencing (RNA-seq), have emerged as powerful tools for investigating the molecular basis of complex traits such as marbling and tenderness. In beef cattle, transcriptomic studies have identified numerous biological pathways—including those related to muscle development, MAPK signaling, insulin/IGF-I signaling, and immune responses—that are enriched with differentially expressed genes (DEGs) in well-marbled versus lean muscle [4,5]. In contrast, transcriptomic studies in sheep remain relatively limited. One recent analysis of sheep liver tissue revealed differential expression of metabolic and stress-response genes between tender and tough meat groups [6], but comprehensive muscle- and fat-specific transcriptomic analyses in sheep are scarce.

Comparative multi-species analyses of meat quality at the transcriptome level are still limited. While some studies have examined gene expression in species such as cattle, pigs, and sheep [7], few have directly compared beef and lamb with respect to carcass quality and tenderness. Such comparisons are valuable for identifying conserved and species-specific molecular mechanisms that influence meat quality and for informing breeding and management strategies across livestock species.

The present study aimed to refine our understanding of the molecular correlates of meat tenderness by comparing beef steers and sheep wethers representing a range of carcass fat endpoints. By integrating traditional carcass measurements (e.g., fat thickness and marbling score) with shear force and RNA-seq-based transcriptomic analyses of longissimus muscle and subcutaneous adipose tissue, we sought to identify key genes and biological pathways associated with variation in meat quality. This work builds on prior preliminary RNA-seq findings in beef steers [8] and contributes to a growing body of research exploring the genomic basis of economically important traits in livestock. Our results provide a comparative perspective on transcriptomic signatures associated with carcass quality in beef and sheep and identify candidate molecular pathways correlated with variation in meat quality traits that merit future functional validation.

## 2. Materials and Methods

### 2.1. Animals and Experimental Design

This study utilized fifteen Angus-cross beef steers and fifteen Columbia-cross wethers (castrated male sheep) to evaluate transcriptomic and phenotypic differences associated with carcass quality. Animals were managed at Montana State University facilities using species-appropriate finishing practices. Beef steers were group-fed a high-energy corn-based finishing diet designed to promote carcass fat deposition, whereas sheep wethers were maintained on a high-quality alfalfa-based diet offered ad libitum. Animals had continuous access to fresh water and were monitored routinely for health and welfare throughout the feeding period. Within each species, animals were managed similarly apart from differences in harvest endpoint associated with target USDA quality grade or Fat Class. These nutritional regimes were selected to promote fat deposition in both species, consistent with previous studies on marbling and meat quality [4,9].

Beef and sheep harvest endpoints were selected prospectively using live-animal visual appraisal of finish, growth rate and body condition in combination with body weight and expected carcass composition. No ultrasound measurements were used to assign animals to harvest endpoint groups. Beef steers were harvested at endpoints corresponding to USDA Standard, Select, and low Choice quality grades (*n* = 5 per group), while sheep were harvested to represent three Fat Classes (Fat Class 1 = lean, Fat Class 2 = intermediate, Fat Class 3 = high fat; *n* = 5 per group). Sheep were initially assigned to Fat Classes based on expected carcass composition, with the goal of five animals per class. However, one sheep carcass was fatter than anticipated at harvest and was reassigned to the appropriate Fat Class based on measured backfat thickness, resulting in final group sizes of 4–6 animals per class. Final carcass classification was confirmed post-harvest using measured carcass traits. Beef carcasses were classified according to USDA quality grade based on marbling and maturity, whereas sheep carcasses were assigned to Fat Class based on measured carcass fatness, including backfat thickness. This design was intended to capture a range of marbling and fatness phenotypes, similar to prior transcriptomic studies in livestock [6,7]. All animal procedures were approved by the Montana State University Agricultural Animal Care and Use Committee (protocol #2019-AA08).

### 2.2. Carcass Trait and Tenderness Measurements

**Figure 1:**
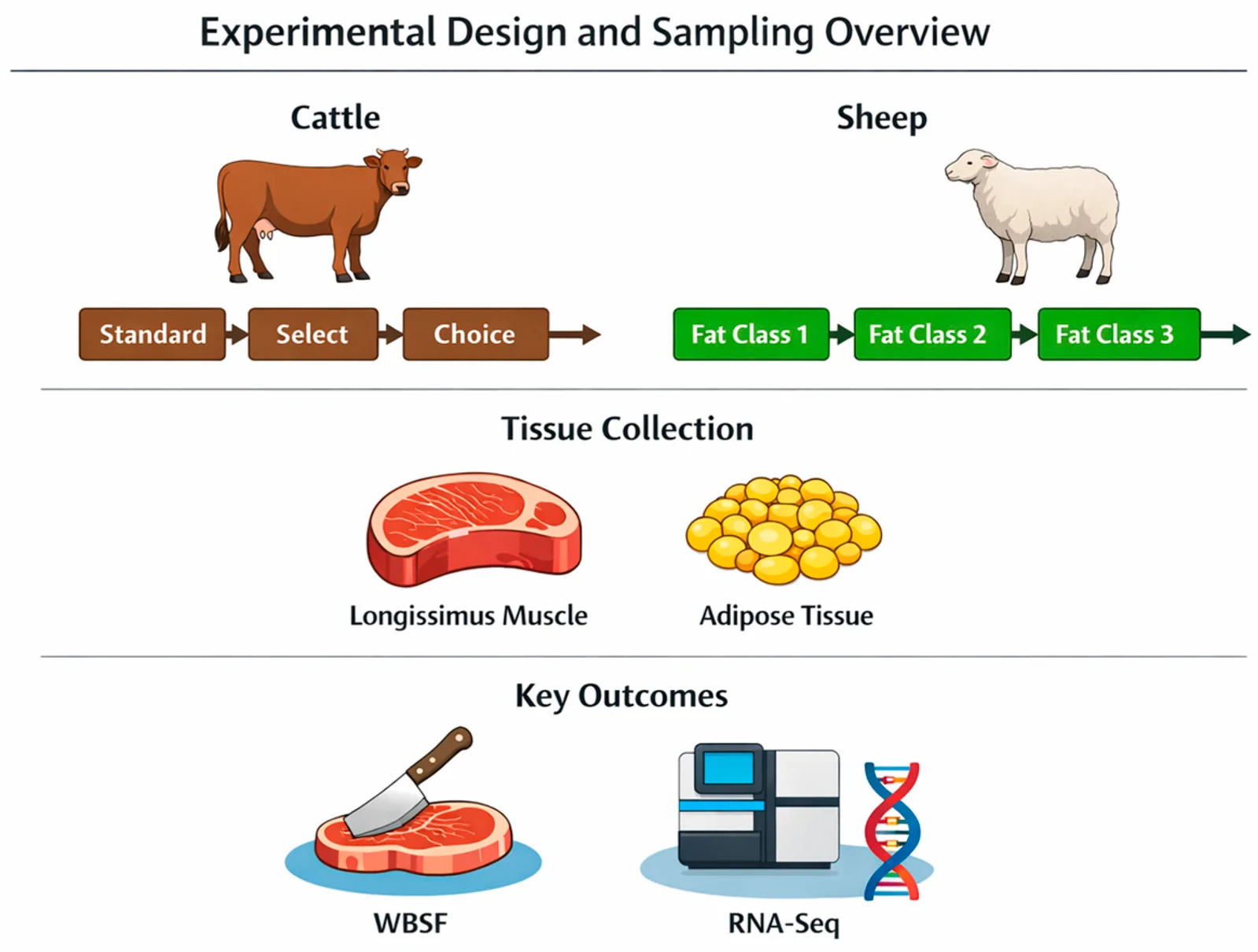
Experimental design and sample schematic showing the species included, tissues sampled, and key outcomes measured in this study.

Standard carcass traits were recorded at harvest, including hot carcass weight (HCW), backfat thickness, ribeye area (longissimus muscle area), marbling score (beef), and flank fat streaking (sheep). To assess tenderness, one 2.54 cm thick Longissimus lumborum steak was collected from each carcass and aged under refrigeration for 14 days before tenderness evaluation, then cooked to an internal temperature of 70 °C using a standardized broiling protocol. Six to eight cores (1.27 × 1.27 × 2.54 cm) were excised parallel to muscle fibers, and shear force was measured using a TMS 30 Food Texturometer (Food Technology Corporation, Sterling, VA, USA) fitted with a Warner–Bratzler blade supplied with the TMS 30 Food Texturometer system (Food Technology Corporation, Sterling, VA, USA). The mean peak shear force (N) per steak was recorded, with lower values indicating greater tenderness [1,10]. A graphical overview of the experimental design, species, tissues sampled, and analytical workflow is provided in Figure 1.

### 2.3. Tissue Collection and RNA Extraction

At harvest, approximately 3 g of longissimus muscle and subcutaneous adipose tissue were collected from each carcass, flash-frozen in liquid nitrogen, and stored at −80 °C. Total RNA was extracted using the Qiagen RNeasy Plus Universal Mini Kit (Qiagen, Hilden, Germany), following the manufacturer’s protocol. RNA quality and concentration were assessed using a NanoDrop spectrophotometer (Thermo Fisher Scientific, Waltham, MA, USA) and Agilent 2100 Bioanalyzer (Agilent Technologies, Santa Clara, CA, USA). Only samples with RNA integrity number (RIN) ≥ 8.0 were used for sequencing.

### 2.4. RNA Sequencing and Data Processing

RNA-seq libraries were prepared using the Illumina TruSeq Stranded mRNA Library Prep Kit (Illumina, Inc., San Diego, CA, USA) and sequenced on an Illumina NovaSeq 6000 platform (Illumina, Inc., San Diego, CA, USA) to generate 150 bp paired-end reads. Raw reads were quality-checked using FastQC (Babraham Bioinformatics, Cambridge, UK; https://www.bioinformatics.babraham.ac.uk/projects/fastqc/; accessed on 30 July 2026) [11] and trimmed with Trimmomatic (http://www.usadellab.org/cms/?page=trimmomatic; accessed on 30 July 2026) [12]. Reads were aligned to the Bos taurus ARS-UCD1.2 and Ovis aries Oar_v4.0 reference genomes using the STAR aligner (https://github.com/alexdobin/STAR; accessed on 30 July 2026) [13]. Gene-level read counts were quantified using featureCounts (Subread package; https://subread.sourceforge.net/featureCounts.html; accessed on 30 July 2026) [14]. Raw integer count matrices were retained for differential expression analysis with DESeq2 (Bioconductor; https://bioconductor.org/packages/DESeq2/; accessed on 30 July 2026). TPM values were calculated for descriptive summaries and cross-sample expression visualization, but TPM-normalized values were not used for statistical testing of differential expression. Expression distribution quality was assessed using log_2_(FPKM + 1) density plots across all samples (Supplementary Figure S1), and these FPKM-derived values were used only for visualization and quality-control purposes. Sample-to-sample Pearson correlation heatmaps confirmed consistency across replicates and tissues (Supplementary Figure S2).

RNA-seq quality metrics supported downstream analysis across retained libraries. In beef libraries, clean read depth ranged from 19.02 to 30.40 million reads per sample, with Q20 values of 97.58–98.32%, Q30 values of 93.13–95.03%, and GC content of 49.11–54.74%. STAR alignment showed total mapping rates of 87.39–94.93%, uniquely mapping rates of 84.64–91.55%, and multiple-mapping rates of 1.65–3.53%. In sheep libraries, clean read depth ranged from 19.96 to 23.60 million reads per sample, with Q20 values of 98.08–98.31%, Q30 values of 94.42–94.94%, and GC content of 49.14–52.03%. Sheep total mapping rates ranged from 87.52 to 91.00% in muscle and 89.06–89.73% in adipose tissue, with multiple-mapping rates of 8.38–22.95%. Gene-assigned read totals calculated by summing the featureCounts gene-level count matrix ranged from 12.96 to 22.02 million reads per beef library and 6.62 to 14.58 million reads per sheep library.

### 2.5. Differential Expression and Functional Enrichment Analysis

Differential gene expression analysis was performed using DESeq2 (Bioconductor; https://bioconductor.org/packages/DESeq2/; accessed on 30 July 2026) [15] with raw gene-level count data as input and DESeq2 normalization and dispersion estimation applied internally. TPM and FPKM values were not used for differential expression testing. Genes with a false discovery rate (FDR)-adjusted *p*-value < 0.05 and absolute log_2_ fold-change ≥ 1.0 were considered significantly differentially expressed. Principal component analysis (PCA) was conducted to assess global transcriptomic variation. Functional enrichment analysis of DEGs was performed using DAVID Bioinformatics Resources (https://davidbioinformatics.nih.gov/; accessed on 30 July 2026) [16], and KEGG pathway analysis was used to identify overrepresented biological processes and signaling pathways. Protein–protein interaction (PPI) networks were constructed using the STRING database (version 12.0; https://string-db.org/; accessed on 30 July 2026) [17]. Pathway enrichment analyses presented in Supplementary Figures S4–S7 were performed using Ingenuity Pathway Analysis (IPA; QIAGEN, Hilden, Germany; https://digitalinsights.qiagen.com/products-overview/discovery-insights-portfolio/analysis-and-visualization/qiagen-ipa/; accessed on 30 July 2026), which identifies overrepresented canonical pathways based on curated biological knowledge. These results complement the KEGG and GO analyses by providing additional mechanistic insights.

### 2.6. Statistical Analysis of Phenotypic Data

Carcass and shear force data were analyzed using one-way analysis of variance (ANOVA) to assess differences among USDA quality grades (beef) and Fat Classes (sheep). Tukey’s honestly significant difference (HSD) test was used for post hoc pairwise comparisons when the overall ANOVA was significant. Statistical significance was set at α = 0.05. Although each sheep Fat Class was initially designed to include five animals, one carcass was fatter than expected at harvest and was reassigned to the appropriate Fat Class based on measured backfat thickness, resulting in final group sizes of 4–6 animals. Animals were selected for inclusion to minimize age-related variation, with similar birth dates and weaning weights within species. However, because the study used an endpoint-based design with limited sample size, body weight, growth rate, and carcass endpoint were not included as covariates in the primary models; therefore, potential confounding among growth, carcass fatness, and tenderness cannot be fully excluded. All statistical analyses were conducted using R version 4.2.2 (R Foundation for Statistical Computing, Vienna, Austria) [18].

## 3. Results

### 3.1. Carcass Traits and Meat Tenderness

**Figure 2:**
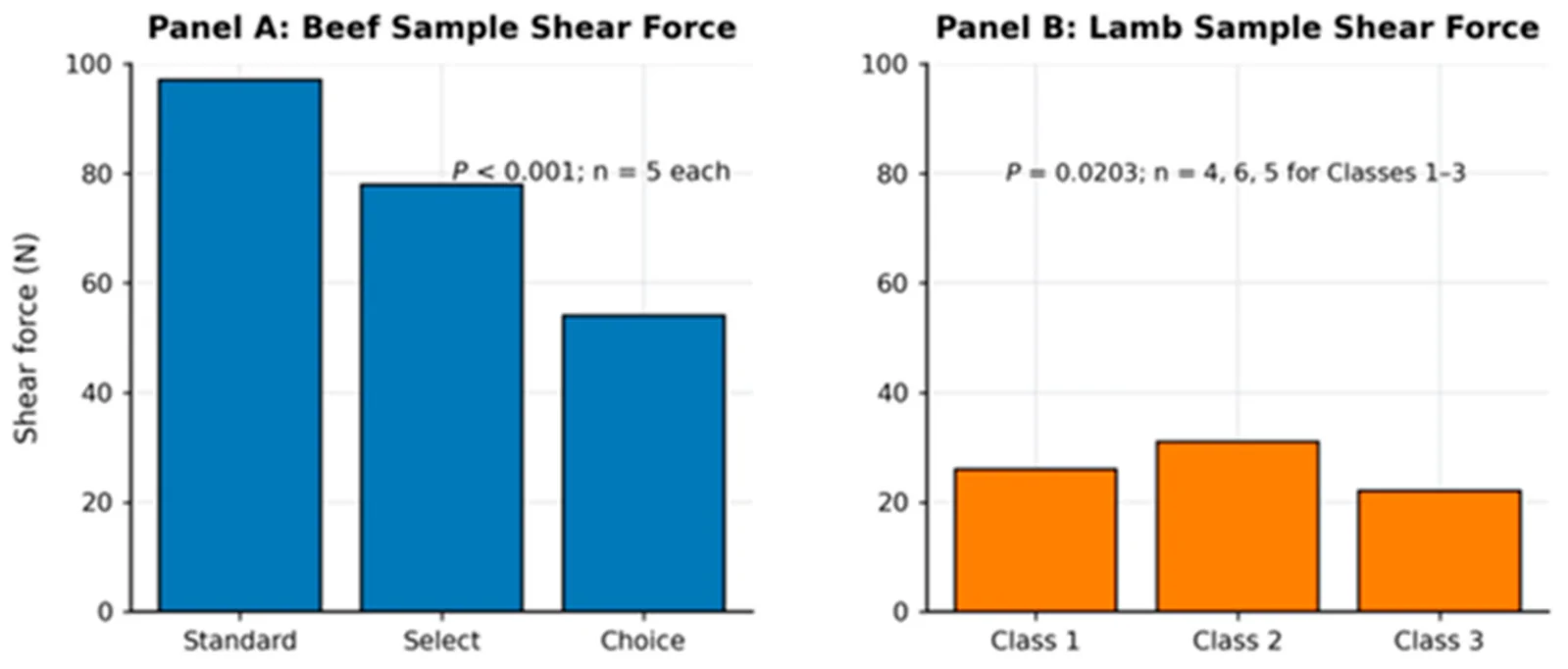
Shear force values for longissimus steaks from (**A**) beef carcasses stratified by USDA quality grade (Standard, Select, Choice) and (**B**) sheep carcasses stratified by Fat Classes (Fat Class 1–3). In beef, shear force differed significantly across quality grades (*p* < 0.0001; *n* = 5 per group), with mean shear force values of 96.51 N (Standard), 77.41 N (Select), and 54.22 N (Choice). In sheep, numerical mean shear force varied among Fat Classes (ANOVA *p* = 0.0203), with mean values of 25.64 N (Fat Class 1), 31.59 N (Fat Class 2), and 22.21 N (Fat Class 3). Because the pattern was non-monotonic and group sizes were limited, Fat Class should not be interpreted as a reliable predictor of lamb tenderness.

The experimental design successfully generated divergence in carcass fatness and quality between the two species. In sheep, animals in the highest Fat Class (Fat Class 3) exhibited significantly greater hot carcass weight (HCW) and backfat thickness compared to leaner Fat Class 1 wethers (*p* < 0.05), although ribeye area and average daily gain did not differ significantly across Fat Classes. In beef, steers graded as USDA Choice demonstrated significantly higher average daily gain, backfat thickness, ribeye area, and marbling scores compared to those graded as Select or Standard (*p* < 0.05), confirming the intended stratification of carcass quality (Figure 2A,B). Because average daily gain also differed across beef quality grade groups, it is possible that some of the observed differences in carcass traits reflect, in part, variation in growth rate rather than carcass grade alone.

Shear force testing confirmed that beef steaks from higher-grade carcasses were more tender: Choice-grade steaks had significantly lower shear force values than Standard-grade steaks (*p* < 0.05), consistent with expectations that increased marbling improves tenderness (Figure 2A). In sheep, numerical shear force means varied among Fat Classes (Figure 2B). Mean shear force values were 25.64 N for Fat Class 1, 31.59 N for Fat Class 2, and 22.21 N for Fat Class 3. Although the overall ANOVA indicated variation among group means (*p* = 0.0203), the pattern was not monotonic and should be interpreted cautiously given the limited sample size and the reclassification of one carcass based on backfat thickness. Animals were selected to represent carcass fatness endpoints rather than randomly assigned treatment groups, and body weight, growth rate, and carcass class were not included as covariates. Therefore, these results do not support interpreting Fat Class as a clear or reliable predictor of lamb tenderness.

### 3.2. Global Transcriptomic Structure

**Figure 3:**
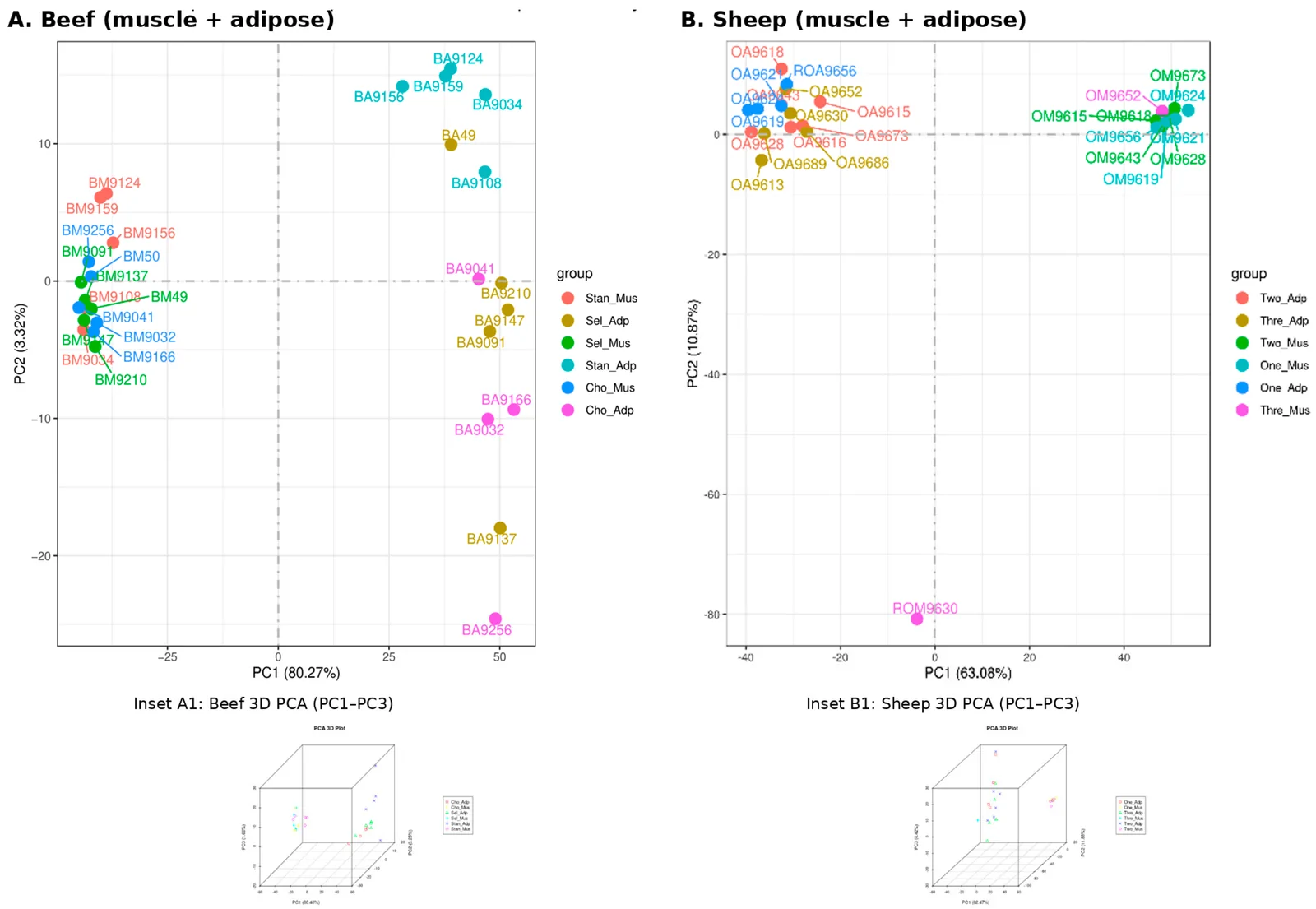
Global transcriptome structure of beef and sheep RNA-seq datasets. Principal component analysis (PCA) of normalized gene expression values summarizes global transcriptomic variation prior to differential expression analysis. (**A**) Beef longissimus muscle and adipose samples and (**B**) sheep muscle and adipose samples are shown as PC1 vs. PC2. Insets show 3D PCA views (PC1–PC3) for each species, visualizing clustering across the three principal components. Detailed RNA-seq quality control visualizations are provided in the Supplementary Materials (Figures S1 and S2).

Principal component analysis (PCA) of normalized gene expression values revealed clear separation by tissue type and species, with distinct clustering of muscle and adipose samples in both cattle and sheep (Figure 3A,B). These patterns were supported by RNA-seq quality control metrics, including consistent expression distributions across samples (Supplementary Figure S1) and high sample-to-sample correlation within tissue and species groups (Supplementary Figure S2). These results confirm the technical robustness of the RNA-seq data and justify downstream differential expression analyses.

### 3.3. Differential Gene Expression

**Figure 4:**
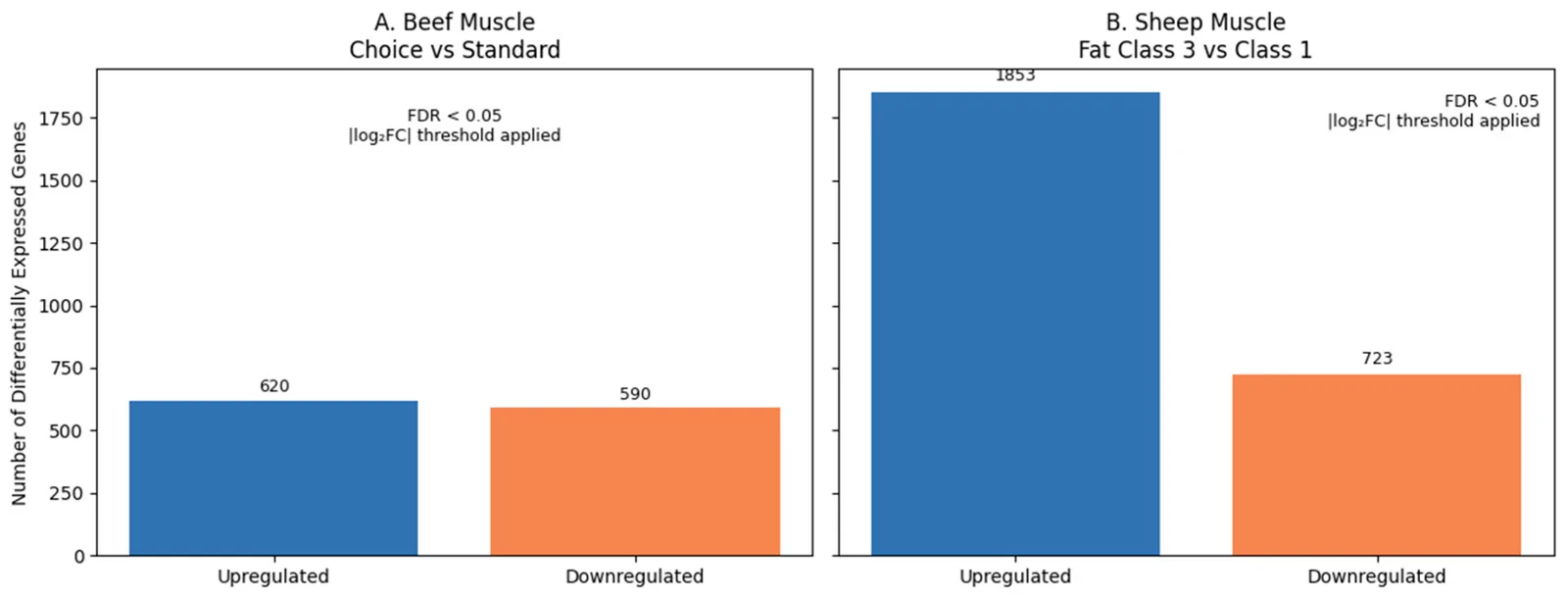
Differential expression magnitude and direction in beef and sheep longissimus muscle. Bar plots summarize the number of significantly up- and down-regulated genes identified by RNA-seq in (**A**) beef longissimus muscle comparing Choice vs. Standard quality grade and (**B**) sheep longissimus muscle comparing Fat Class 3 vs. Fat Class 1. Differential expression was assessed using an FDR-adjusted threshold (FDR < 0.05) and a minimum absolute log_2_ fold-change cutoff.

Differential expression analysis identified substantial transcriptomic differences between high- and low-USDA quality grade beef carcass groups and high- and low-fat sheep carcasses. In beef muscle, comparisons between Choice and Standard grade carcasses yielded several hundred DEGs, with a relatively balanced distribution of upregulated and downregulated genes (Figure 4A). In contrast, sheep muscle exhibited a much larger number of DEGs: 1853 genes were upregulated and 723 downregulated in high-fat (Fat Class 3) versus low-fat (Fat Class 1) carcasses (Figure 4B). These results are consistent with more extensive transcriptional differences associated with sheep Fat Class in muscle; however, given the limited sample size, final group imbalance, and lack of clear separation in lamb shear force, the magnitude of this DEG response should be interpreted cautiously. The larger DEG set in sheep should be viewed as evidence of Fat Class-associated transcriptomic remodeling rather than evidence of a proportional or validated effect on tenderness.

Volcano plots illustrating the magnitude and significance of DEGs across species and tissues are provided in Supplementary Figure S3. These plots, together with the pathway enrichment results in Supplementary Figure S6, support an exploratory pattern of broad transcriptomic remodeling in sheep muscle, particularly in animals classified in the higher sheep Fat Class group. Enriched pathways included glycolysis, gluconeogenesis, and interferon signaling, which may reflect coordinated metabolic and immune differences associated with carcass fatness rather than validated mechanisms of lamb tenderness.

### 3.4. Functional Enrichment and Pathway Analysis

**Figure 5:**
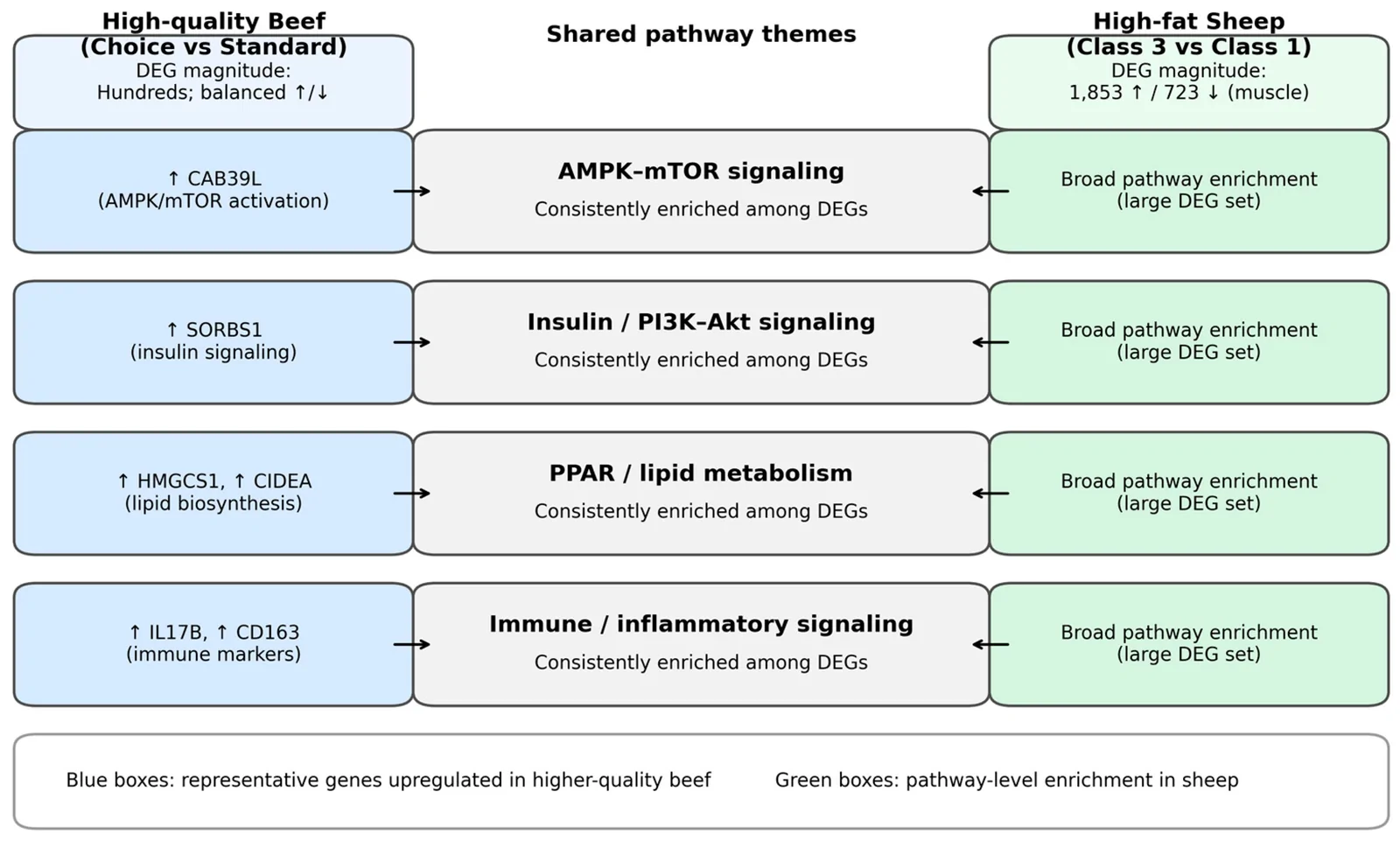
Key pathways altered with carcass quality and fatness across species. Conceptual synthesis of pathway-level enrichment themes observed in muscle and adipose RNA-seq comparisons. Four recurring signaling themes are highlighted: AMPK–mTOR, insulin/PI3K–Akt, PPAR/lipid metabolism, and immune/inflammatory signaling. Example genes upregulated in higher-quality beef are shown as representative markers within each theme. The sheep comparison exhibits broad enrichment across these same themes in the context of a substantially larger DEG set.

Functional enrichment analysis revealed that DEGs in both species were significantly enriched in pathways related to muscle development, energy metabolism, adipogenesis, and immune/inflammatory signaling. In beef muscle, upregulated genes in higher-grade carcasses included CAB39L (an activator of AMPK signaling) and SORBS1 (involved in insulin signaling), while DTX4 (a Notch pathway component) was downregulated (Figure 5). In adipose tissue, genes such as HMGCS1 (cholesterol biosynthesis) and CIDEA (lipid droplet formation) were upregulated in higher-grade beef, whereas NRIP3 (nuclear receptor-interacting protein 3, a transcriptional co-regulator also known as C11orf14) was strongly downregulated, suggesting shifts in lipid metabolism and hormonal signaling. Notably, the most significantly enriched pathway in beef adipose tissue was ‘Neutrophil Degranulation’ (−log_10_(p) = 18.0; Supplementary Figure S5), highlighting a robust immune response in high-grade carcasses. This finding supports the observed upregulation of immune-related genes such as IL17B and CD163 and suggests substantial immune cell infiltration and remodeling in marbled adipose tissue.

In sheep, the extensive DEG profiles in both muscle and adipose tissues were similarly enriched in pathways associated with lipid metabolism, energy regulation, and immune responses. However, these molecular differences should be interpreted as Fat Class-associated transcriptomic variation rather than evidence of a direct tenderness mechanism, because lamb shear force did not show a clear or consistent response to Fat Class in this dataset. Interestingly, several muscle-related pathways, including ‘Striated Muscle Contraction’ and ‘ILK Signaling,’ were among the top-enriched pathways in sheep adipose tissue (Supplementary Figure S7). This may reflect cross-tissue gene expression or the presence of muscle-associated transcripts in adipose depots of high-fat sheep, potentially linked to vascularization or connective tissue remodeling.

**Figure 6:**
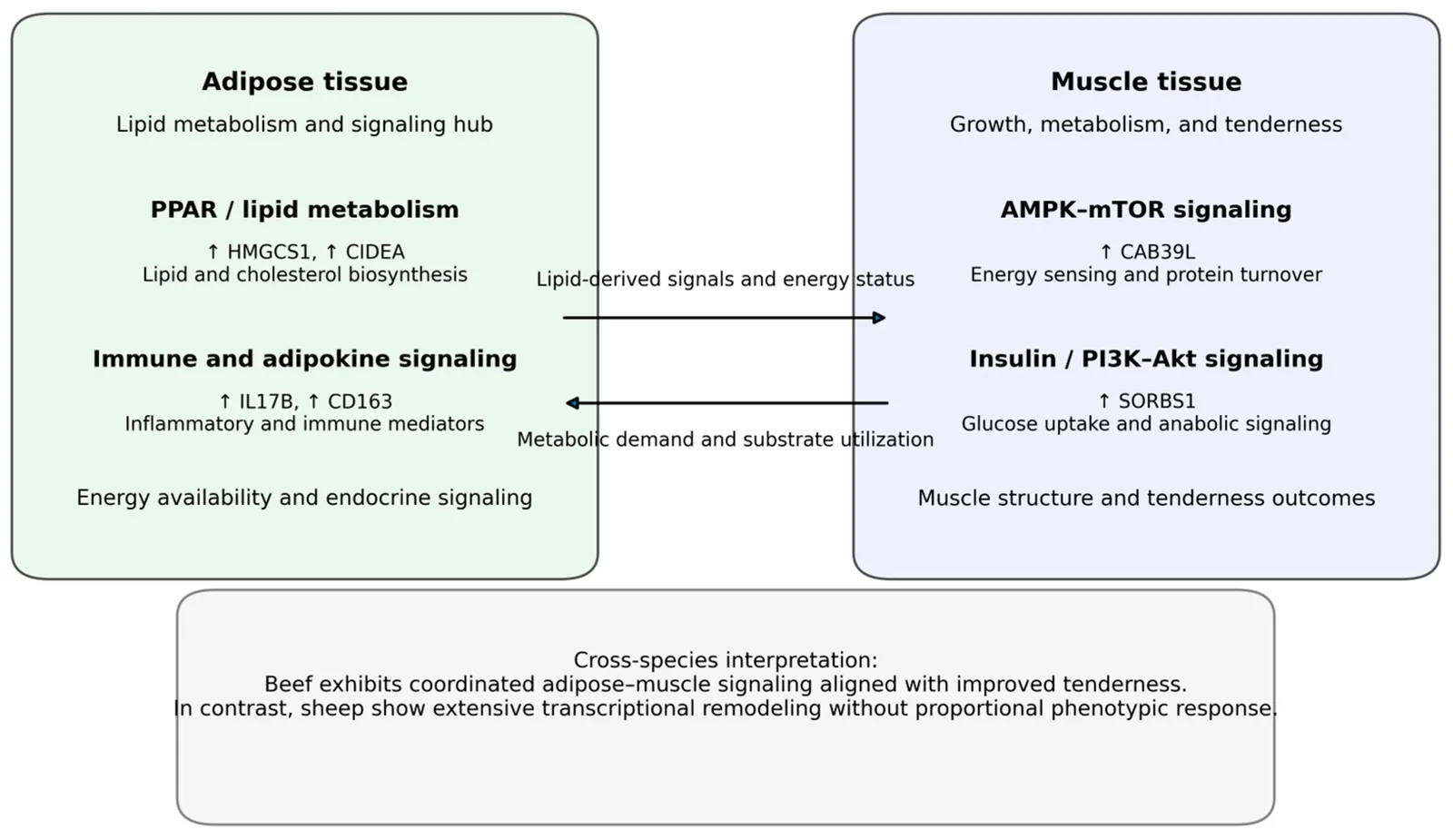
Muscle–adipose cross-tissue integration underlying meat quality. Conceptual model integrating transcriptomic signals from adipose and muscle tissues. Adipose tissue exhibits altered lipid metabolism, immune, and endocrine signaling pathways associated with carcass quality or fatness, generating lipid-derived and energetic signals that influence muscle energy sensing, insulin signaling, and protein turnover. In beef, these pathways appear to be coordinated with lower shear force in higher-grade carcasses, whereas in sheep, extensive transcriptional remodeling across tissue occurred without clear evidence that Fat Class was a reliable predictor of lamb tenderness.

Detailed pathway enrichment results for each tissue and species are provided in Supplementary Figures S4–S7. These include dot plots and ranked pathway tables that support the pathway-level summaries presented in Figure 5 and the cross-tissue integration model in Figure 6.

### 3.5. Cross-Tissue Integration

A conceptual model integrating transcriptomic signals from muscle and adipose tissues is presented in Figure 6. In beef, coordinated upregulation of genes involved in anabolic growth, energy metabolism, and lipid biosynthesis in both tissues was associated with improved tenderness in higher-grade carcasses. In sheep, although similar pathways were enriched, the absence of a clear Fat Class-related tenderness response suggests that these transcriptomic differences may reflect fat deposition, physiological maturity, tissue remodeling, or production-system effects rather than tenderness per se. These findings support the hypothesis that cross-tissue signaling between adipose and muscle may be associated with meat quality, particularly in beef, but this hypothesis requires validation through larger studies and functional approaches.

## 4. Discussion

This comparative analysis of beef cattle and sheep provides new insights into the molecular and phenotypic correlates of meat quality, particularly tenderness and carcass fatness. In beef, higher USDA quality grades (Select/Choice vs. Standard) were associated with significantly lower shear force values, consistent with industry expectations that increased marbling improves tenderness [1,2]. The beef results are broadly consistent with prior reports showing that marbling and USDA quality grade are associated with improved eating quality, although marbling alone does not fully explain tenderness variation. Warner et al. [1] emphasized that tenderness is influenced by multiple biological factors, including intramuscular fat, connective tissue characteristics, postmortem proteolysis, and muscle structure. The role of connective tissue, particularly collagen content and cross-linking, as a contributor to meat tenderness has also been reviewed extensively [19]. Rodriguez et al. [2] similarly reported that carcass and meat quality traits are related but imperfect predictors of shear force, while Vierck et al. [3] showed that marbling texture can influence palatability without fully accounting for Warner–Bratzler shear force variation. Recent national tenderness survey data further indicate that shear force and consumer tenderness ratings vary across product categories and market sources, and that USDA quality grade effects are not always uniform across all beef products [20]. Together, these findings support the direction of the beef results observed here while reinforcing that marbling and quality grade should be interpreted as contributors to tenderness rather than sole determinants.

In sheep, Fat Class was neither a clear nor a reliable predictor of lamb tenderness in this dataset. Although numerical shear force means varied among Fat Classes, the pattern was not monotonic and should not be interpreted as evidence that increasing external fatness consistently improves tenderness. This distinction is important because sheep Fat Class primarily reflects carcass fatness, including external or subcutaneous fat, whereas eating quality is influenced by multiple traits, including intramuscular fat, muscle fiber characteristics, connective tissue properties, chilling rate, and postmortem proteolysis. Thus, the sheep results are better interpreted as showing that Fat Class was associated with transcriptomic differences in muscle and adipose tissue, but those molecular differences did not correspond to a clear tenderness advantage. This interpretation is consistent with prior lamb meat-quality literature indicating that external fatness, intramuscular fat, and tenderness are related but distinct traits. Pannier et al. [21] noted that lamb eating quality is affected by multiple production, carcass, and processing factors and that grading systems may need to integrate traits beyond fatness alone. Realini et al. [22] reported that intramuscular fat level can influence consumer liking of pasture-finished lamb, whereas Fat Class or external fatness may not directly capture the intramuscular or structural traits most closely linked to eating quality. Similarly, Zhang et al. [23] described intramuscular fat as an important contributor to meat quality and discussed regulatory mechanisms influencing intramuscular fat deposition in Tan sheep.

Several biological and classification-system differences may explain why USDA quality grade was more strongly associated with tenderness in beef than Fat Class was in sheep. USDA quality grade in beef directly incorporates marbling, which reflects intramuscular fat deposition within the longissimus muscle and is closely linked to juiciness, flavor, and, to a lesser extent, tenderness. In contrast, sheep Fat Class primarily reflects carcass fatness, including external or subcutaneous fat, and may not closely track intramuscular fat, muscle fiber characteristics, connective tissue properties, or postmortem proteolysis. Sheep also differ from cattle in fat-partitioning patterns and are generally harvested at younger physiological maturity, when baseline tenderness may already be relatively high and additional fat deposition may produce only limited reductions in shear force. Therefore, the weaker and non-linear relationship between Fat Class and tenderness in sheep likely reflects both biological differences in fat deposition and the fact that Fat Class is not directly equivalent to beef marbling or USDA quality grade.

An additional explanation is that sheep samples may have been within a relatively tender baseline range, producing a potential floor effect for shear force. The mean sheep shear force values in this study ranged from 22.21 to 31.59 N, substantially lower than the beef means of 54.22 to 96.51 N. Thus, additional fat deposition in sheep may have had limited opportunity to further reduce shear force, even when accompanied by broad transcriptomic differences. For this reason, the sheep transcriptomic results should not be interpreted as biologically ineffective; rather, they may reflect molecular variation occurring within an already tender phenotypic range.

An additional consideration when interpreting cross-species differences is the contrasting histories of selection and the industry’s emphasis on carcass-quality traits. In beef cattle, genetic selection, value-based marketing, and carcass grading have placed substantial emphasis on marbling and eating-quality traits. In sheep production systems, selection pressure has more often emphasized growth, leanness, reproductive performance, and carcass yield, while marbling is not routinely measured or used as a primary grading trait. Sheep are also typically harvested at a younger physiological age than cattle, which may limit variation in collagen cross-linking and baseline tenderness relative to older beef carcasses. Therefore, sheep Fat Class may capture carcass finish or chilling protection more than intramuscular fat, connective tissue, and postmortem traits most closely associated with tenderness. This distinction may help explain why sheep exhibited extensive differential gene expression across Fat Classes without a clear corresponding tenderness response.

Dietary differences between species should also be considered when interpreting the comparative transcriptomic results. Beef steers were finished on a high-energy corn-based diet, whereas sheep were maintained on a high-quality alfalfa-based diet; therefore, species and diet effects cannot be fully separated in the present design. Some of the interspecies transcriptomic differences may reflect differences in dietary starch, fiber, protein, energy density, rumen fermentation patterns, or metabolic state rather than intrinsic species-specific regulation of meat quality alone. Consequently, the cross-species transcriptomic comparisons should be interpreted as reflecting the combined influence of species, production system, diet, and carcass endpoint. Whether feeding sheep a higher-energy or higher-protein diet would shift their transcriptomic profile or strengthen the relationship between fatness and tenderness remains an open question that would require a controlled feeding study with diet balanced across species or varied within species.

Transcriptomic analyses revealed substantial differences in gene expression between high- and low-quality carcass groups in both species. Notably, sheep muscle exhibited a markedly larger number of differentially expressed genes (DEGs) than beef muscle, particularly in comparisons between high-fat (Fat Class 3) and low-fat (Fat Class 1) carcasses. These extensive transcriptional changes in sheep muscle and adipose tissue were not mirrored by a clear Fat Class-related tenderness advantage, raising questions about the functional relevance of these molecular differences. One plausible explanation is that fatness in sheep may reflect subcutaneous or intermuscular fat accumulation rather than intramuscular marbling, which is more directly linked to tenderness. Previous studies have shown that intramuscular fat (IMF) is positively associated with lamb eating quality, including tenderness, while subcutaneous fat has a weaker or inconsistent relationship with sensory traits [21,22]. This distinction is important because species differ in patterns of fat deposition and fat partitioning, and sheep may deposit external or intermuscular fat without equivalent increases in intramuscular fat [24]. Therefore, the sheep Fat Class results should not be interpreted as directly analogous to USDA quality grade or marbling effects in beef, because the two systems may reflect different patterns of fat deposition and fat partitioning. Additionally, factors such as muscle fiber type composition, connective tissue content, and postmortem proteolytic activity—known contributors to tenderness—were not directly assessed in this study but may play a more dominant role in sheep meat quality [1,25].

The larger DEG set observed in sheep muscle may represent a broad transcriptomic remodeling response to fatness, diet, growth state, or tissue adaptation rather than a proportional mechanism for tenderness improvement. In this context, the apparent “transcriptome over-response” may reflect coordinated changes in metabolic, immune, extracellular matrix, and stress-response pathways associated with fat deposition and physiological adaptation. DEG number alone should therefore not be interpreted as evidence of a stronger effect on meat quality, particularly because Fat Class did not provide a clear or reliable predictor of lamb tenderness in this dataset. The disconnect between extensive transcriptional differences and the absence of a clear Fat Class-related tenderness response in sheep may also indicate that post-transcriptional regulation, protein turnover, muscle fiber composition, collagen characteristics, or postmortem proteolysis have greater influence on tenderness than transcript abundance alone.

In both species, DEGs were enriched in pathways related to muscle development (e.g., AMPK–mTOR signaling), lipid metabolism (e.g., PPAR signaling), and immune/inflammatory responses. These findings align with previous transcriptomic studies in beef cattle that have implicated similar biological processes in marbling and meat quality [4,5]. For example, the upregulation of CAB39L and SORBS1 in high-grade beef muscle suggests enhanced energy metabolism and insulin signaling, while the downregulation of DTX4 may reflect reduced Notch pathway activity, potentially favoring muscle fiber development. Although CAB39L, SORBS1, and DTX4 are biologically plausible candidate genes because of their roles in energy metabolism, insulin signaling, and Notch-related developmental regulation, respectively, they should not yet be considered validated molecular markers for beef tenderness. Their potential utility would require confirmation of consistent expression–phenotype associations in independent beef populations, targeted validation by qPCR or digital PCR, and integration with genotype, protein-abundance, and tenderness phenotypes. Because these signaling pathways are broadly conserved across mammals, related functions may be present in sheep; however, conservation of their specific relationship with tenderness has not been demonstrated and should be tested directly before cross-species marker use is considered. In adipose tissue, the upregulation of HMGCS1 and CIDEA in higher-grade beef supports a role for lipid biosynthesis and storage in marbling-associated gene expression profiles. The observed upregulation of immune-related genes, such as IL17B and CD163, in adipose tissue from higher-grade carcasses may reflect increased immune cell infiltration or inflammatory signaling, a phenomenon previously linked to adipose remodeling in livestock and to inflammation -associated metabolic remodeling in mammalian tissues [24,26].

In both species, transcriptomic analyses revealed that carcass quality and fatness were associated not only with metabolic and structural remodeling but also with broader shifts in tissue signaling environments. In particular, adipose tissue from higher-quality beef carcasses exhibited transcriptional signatures indicative of dynamic tissue remodeling, including pathways related to cellular trafficking, extracellular matrix organization, and intercellular communication. These changes may reflect an adaptive response to increased lipid accumulation and metabolic demand, potentially involving interactions between stromal and immune cells. Similarly, high-fat sheep tissues showed coordinated activation of signaling networks involved in stress response and tissue adaptation, suggesting that increased fatness may elicit systemic physiological adjustments beyond lipid storage. While the functional consequences of these responses remain to be fully elucidated, they may contribute to the observed differences in carcass traits and meat quality, particularly in beef.

Because this study included a limited number of animals per group and the final sheep Fat Class groups were slightly unbalanced after post-harvest reclassification, the transcriptomic results should be interpreted as exploratory and hypothesis-generating rather than confirmatory. Despite these associations, it is important to emphasize that the current study is correlational. The observed differences in gene expression cannot be assumed to cause the phenotypic variation in tenderness or carcass quality. Moreover, the small sample size (*n* = 5 per group) limits the statistical power of the analysis and increases the risk of false positives, even with appropriate false discovery rate (FDR) correction. Small sample sizes can also increase sensitivity to individual-animal variation and reduce the stability of DEG and pathway-enrichment results, particularly when final group sizes are uneven. Future studies with larger cohorts and functional validation—such as gene knockdown or overexpression experiments—are needed to confirm the causal roles of the identified genes and pathways. Therefore, the genes and pathways identified here should be viewed as molecular associations with carcass quality or fatness classes, not as validated causal mechanisms of tenderness.

The conceptual model presented in Figure 6 proposes a cross-tissue integration of muscle and adipose transcriptomic signals that may influence meat quality. In beef, the coordinated upregulation of metabolic and anabolic pathways in both tissues was associated with improved tenderness in higher-grade carcasses. In sheep, however, the lack of a clear phenotypic response despite extensive transcriptomic changes suggests that additional regulatory mechanisms or tissue-specific factors may modulate the relationship between fatness and tenderness. These findings support the hypothesis that cross-tissue signaling between adipose and muscle may influence meat quality, particularly in beef, though larger independent studies and functional validation are needed before these interactions can be interpreted as causal.

From a production perspective, these findings do not directly support adopting a beef-style marbling or USDA quality-grade framework for lamb without additional validation. Rather, they suggest that sheep meat-quality assessment may require indicators that better capture intramuscular fat, muscle fiber characteristics, connective tissue properties, postmortem proteolytic potential, and processing conditions such as chilling rate. In lamb, external fat may be more relevant as a buffer against rapid chilling and cold shortening than as a direct indicator of marbling-driven tenderness, which may help explain why Fat Class alone was not a reliable predictor of eating quality in this dataset. For beef, the results support the continued value of carcass quality grade as a practical indicator of eating quality, while also reinforcing that tenderness is multifactorial and may benefit from integration with validated molecular or proteomic markers in the future. However, because this study was exploratory and based on a limited sample size, no immediate changes to grading or selection systems are recommended from these data alone.

Finally, while this study contributes novel comparative insights, it also highlights the importance of integrating transcriptomic data with other layers of biological information, including proteomics, metabolomics, and histological assessments. Incorporating measures of intramuscular fat content, muscle fiber type, and postmortem proteolytic enzyme activity (e.g., calpains and calpastatin) would provide a more comprehensive understanding of the biological determinants of tenderness. Targeted validation approaches, including qPCR confirmation of candidate genes and independent replication in larger populations, will be necessary to determine whether the transcriptomic patterns observed here are repeatable and biologically meaningful. Repeatability of key DEGs should be evaluated by prioritizing genes with consistent expression direction across individual animals, moderate-to-large effect sizes, biological relevance to tenderness or fat deposition, and recurrence across independent cohorts or complementary datasets. Candidate DEGs should then be confirmed using targeted qPCR or digital PCR in the original samples and validated in an independent population before being advanced as robust biomarkers. As genomic technologies continue to advance, multi-omics approaches will be essential for unraveling the complex networks that govern meat quality in livestock [9,27].

## 5. Conclusions

In summary, this exploratory comparative study suggests that carcass quality grade in beef is strongly associated with lower shear force and distinct transcriptomic signatures in both muscle and adipose tissues, consistent with previous findings [1,4]. In sheep, Fat Class was associated with broad transcriptomic differences but was not a clear or reliable predictor of lamb tenderness. These findings highlight species-specific differences in how carcass fatness relates to meat quality and suggest that factors beyond external fat deposition—including intramuscular fat, physiological age, connective tissue characteristics, chilling rate, and postmortem proteolysis—may play a more dominant role in determining lamb eating quality. Because of the limited sample size, final imbalance in the sheep groups, and lack of independent validation, the transcriptomic findings should be interpreted as hypothesis-generating rather than confirmatory.

Future studies should incorporate larger sample sizes, direct measures of carcass composition and fat partitioning, and additional layers of biological data—including proteomics, metabolomics, and histological analyses—to better understand the regulatory networks underlying meat tenderness. Independent replication and targeted validation of candidate genes and pathways will be essential before these transcriptomic results can be translated into selection tools, biomarkers, or production recommendations. Multi-omics approaches and functional validation will be essential for translating transcriptomic insights into practical tools for livestock breeding and meat science [9,27].

## Data Availability

The original data presented in this study are openly available in the Dryad Digital Repository at DOI: 10.5061/dryad.q83bk3jxw. Raw data will be available upon publication in the NCBI SRA at PRJNA1460905 for sheep and PRJNA1462762 for beef.

## Supplementary Materials

The following supporting information can be downloaded at: https://www.mdpi.com/article/10.3390/ani16162493/s1. Figure S1 shows RNA-seq expression distribution quality control using density plots of log2(FPKM + 1) values for all samples, with Panel A presenting ovine muscle and adipose tissue samples across Fat Classes and Panel B presenting bovine muscle and adipose tissue samples across USDA quality grades. Figure S2 presents RNA-seq sample-to-sample Pearson correlation heatmaps for ovine and bovine normalized gene expression values across muscle and adipose tissues. Figure S3 provides volcano plots of differential gene expression analyses for ovine muscle, ovine adipose, bovine muscle, and bovine adipose comparisons. Figure S4 presents pathway enrichment results for beef muscle. Figure S5 presents pathway enrichment results for beef adipose, including enriched canonical pathways grouped into immune/inflammatory, muscle/cytoskeleton, lipid metabolism, and energy/signaling categories. Figure S6 presents pathway enrichment results for sheep muscle, with enriched canonical pathways grouped into immune/inflammatory, muscle development, lipid metabolism, and energy/glucose metabolism categories. Figure S7 presents pathway enrichment results for sheep adipose, with enriched canonical pathways grouped into immune/inflammatory, muscle development, lipid/fat metabolism, energy metabolism, and insulin/PI3K-Akt signaling categories.

## References

[1] Warner R.D., Wheeler T.L., Ha M., Li X., Bekhit A.-E.-D., Morton J.D., Vaskoska R., Dunshea F.R., Liu R., Purslow P.P. (2022). Meat tenderness: Advances in biology, biochemistry, molecular mechanisms, and new technologies. Meat Sci..

[2] Rodriguez E.E., Hamblen H., Flowers S., Leal J.D., Carr C., Scheffler T., Mateescu R.G. (2023). Carcass and meat quality traits in Brangus steers. Transl. Anim. Sci..

[3] Vierck K.R., Gonzalez J.M., Houser T.A., Boyle E.A., O’Quinn T.G. (2018). Marbling texture’s effects on beef palatability. Meat Muscle Biol..

[4] Roudbari Z., Coort S.L., Kutmon M., Eijssen L., Melius J., Sadkowski T., Evelo C.T. (2020). Identification of biological pathways contributing to marbling in skeletal muscle to improve beef cattle breeding. Front. Genet..

[5] Tan Z., Jiang H. (2024). Molecular and cellular mechanisms of intramuscular fat development and growth in cattle. Int. J. Mol. Sci..

[6] Listyarini K., Sumantri C., Rahayu S., Islam M.A., Akter S.H., Uddin M.J., Gunawan A. (2023). Hepatic transcriptome analysis reveals genes, polymorphisms, and molecules related to lamb tenderness. Animals.

[7] Ciecierska A., Ebrahimpour Gorji A., Majewska A., Sadkowski T. (2025). Expression of miRNA in the semitendinosus muscle of cattle breeds with varying intramuscular fat deposition. Genes.

[8] Thomson J.M., Schumacher M.L., Boles J.A. (2022). O43: Utilizing RNA-seq to investigate molecular mechanisms impacting meat quality and carcass characteristics in beef steers. Anim. Sci. Proc..

[9] Guo Y., Li S., Na R., Guo L., Huo C., Zhu L., Shi C., Na R., Gu M., Zhang W. (2024). Comparative transcriptome analysis of bovine, porcine, and sheep muscle using interpretable machine learning models. Animals.

[10] Wheeler T.L., Shackelford S.D., Koohmaraie M. (1997). Standardizing collection and interpretation of shear force and sensory tenderness data. Proc. Recip. Meat Conf..

[11] Andrews S. (2010). FastQC: A Quality Control Tool for High Throughput Sequence Data.

[12] Bolger A.M., Lohse M., Usadel B. (2014). Trimmomatic: A flexible trimmer for Illumina sequence data. Bioinformatics.

[13] Dobin A., Davis C.A., Schlesinger F., Drenkow J., Zaleski C., Jha S., Batut P., Chaisson M., Gingeras T.R. (2013). STAR: Ultrafast universal RNA-seq aligner. Bioinformatics.

[14] Liao Y., Smyth G.K., Shi W. (2014). featureCounts: An efficient general purpose program for assigning sequence reads to genomic features. Bioinformatics.

[15] Love M.I., Huber W., Anders S. (2014). Moderated estimation of fold change and dispersion for RNA-seq data with DESeq2. Genome Biol..

[16] Huang D.W., Sherman B.T., Lempicki R.A. (2009). Systematic and integrative analysis of large gene lists using DAVID bioinformatics resources. Nat. Protoc..

[17] Szklarczyk D., Gable A.L., Nastou K.C., Lyon D., Kirsch R., Pyysalo S., Doncheva N.T., Legeay M., Fang T., Bork P. (2021). The STRING database in 2021: Customizable protein–protein networks, and functional characterization of user-uploaded gene/measurement sets. Nucleic Acids Res..

[18] R Core Team (2022). R: A Language and Environment for Statistical Computing.

[19] Weston A.R., Rogers R.W., Althen T.G. (2002). Review: The role of collagen in meat tenderness. Prof. Anim. Sci..

[20] Gonzalez A.A., Williams E.P., Schwartz T.E., Arnold A.N., Griffin D.B., Miller R.K., Gehring K.B., Brooks J.C., Legako J.F., Carr C.C. (2024). National Beef Tenderness Survey—2022: Consumer Sensory Panel Evaluations and Warner-Bratzler Shear Force of Beef Steaks From Retail and Foodservice. Meat Muscle Biol..

[21] Pannier L., Gardner G.E., O’Reilly R.A., Pethick D.W. (2018). Factors affecting lamb eating quality and the potential for their integration into an MSA sheepmeat grading model. Meat Sci..

[22] Realini C.E., Pavan E., Johnson P.L., Font-i-Furnols M., Craigie C.R., Moon C.D. (2021). Consumer liking of pasture-finished lamb is influenced by intramuscular fat level. Meat Sci..

[23] Zhang X., Liu C., Kong Y., Li F., Yue X. (2022). Effects of intramuscular fat on meat quality and its regulation mechanism in Tan sheep. Front. Nutr..

[24] Liu S., Yang Y., Luo H., Pang W., Martin G.B. (2024). Fat deposition and partitioning for meat production in cattle and sheep. Anim. Nutr..

[25] Leal-Gutiérrez J.D., Rezende F.M., Reecy J.M., Kramer L.M., Peñagaricano F., Mateescu R.G. (2020). Whole genome sequence data provides novel insights into the genetic architecture of meat quality traits in beef. Front. Genet..

[26] Wu H., Ballantyne C.M. (2017). Skeletal muscle inflammation and insulin resistance in obesity. J. Clin. Investig..

[27] Hopkins D.L., Mortimer S.I. (2014). Effect of genotype, gender and age on sheep meat quality and a case study illustrating integration of knowledge. Meat Sci..

