# Supplementary material for: Gene Expression Differences in Muscle and Adipose Tissue Help Explain Variation in Meat Tenderness Across USDA Carcass Grades in Beef and Fat Classes in Sheep
Source: Animals (Basel). 2026 Aug 11;16(16):2493. doi: 10.3390/ani16162493 (PMC13508938; doi:10.3390/ani16162493)
Supplement: Supplementary file 1 [file animals-16-02493-s001.zip › animals-4410267-supplementary.pdf]

Supplementary Figures

Figure S1. RNA-seq expression distribution quality control. Density plots of $\log_2$(FPKM + 1) values for all samples illustrate comparable transcript abundance distributions across tissues and species. **(A)** Ovine muscle and adipose tissue samples across fat classes show similar expression density profiles, indicating consistent library preparation and normalization. **(B)** Bovine muscle and adipose tissue samples across USDA quality grades exhibit comparable FPKM distributions. Together, these plots support the validity of downstream PCA and differential expression analyses.

Panel A – Ovine FPKM density (left → right order)

A9615, A9616, A9618, A9628, A9643, A9673, A9613, A9630, A9653, A9686, A9689,
M9615, M9618, M9628, M9643, M9673, M9619, M9621, M9624, M9656,
A9619, A9621, A9624, A9656, M9652, M9630

Panel B – Bovine FPKM density (left → right order)

M9034, M9108, M9124, M9156, M9159, A9137, A9147, A9091, A9210, A49,
M9137, M9147, M9091, M9210, M49,
A9034, A9108, A9124, A9156, A9159,
M9032, M9041, M9166, M50, M9256,
A9032, A9041, A9166, A9256

Figure S1A. Ovine muscle and adipose FPKM density distributions

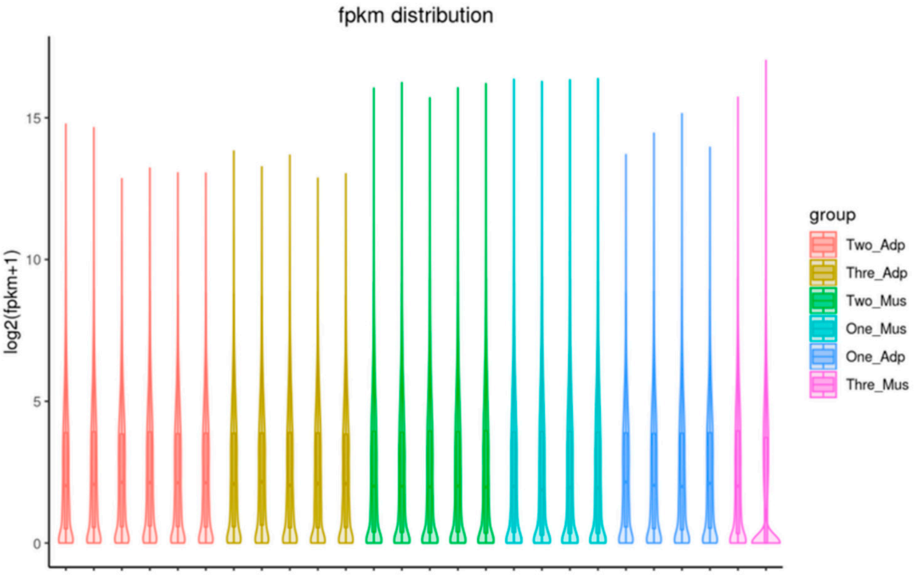

Figure S1B. Bovine muscle and adipose FPKM density distributions

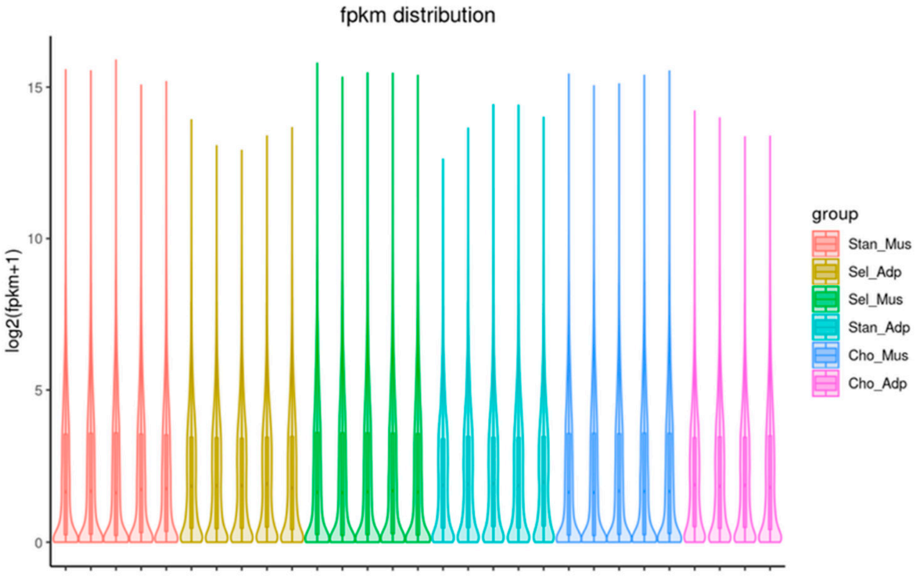

Figure S2. RNA-seq sample-to-sample correlation heatmaps. **(A)** Ovine sample-to-sample Pearson correlation heatmap for normalized gene expression values across muscle and adipose tissues. **(B)** Bovine sample-to-sample Pearson correlation heatmap for normalized gene expression values across muscle and adipose tissues. Darker colors indicate higher correlation between samples, supporting overall RNA-seq consistency prior to downstream analyses.

Figure S2A. Ovine RNA-seq sample correlation heatmap (Pearson)

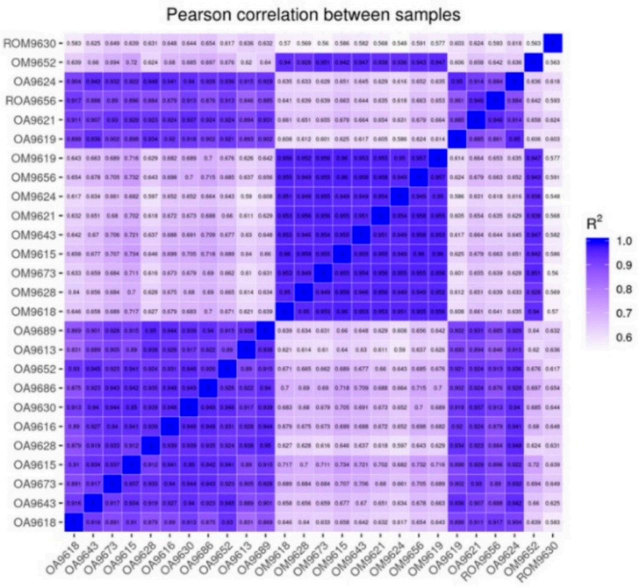

Figure S2B. Bovine RNA-seq sample correlation heatmap (Pearson)

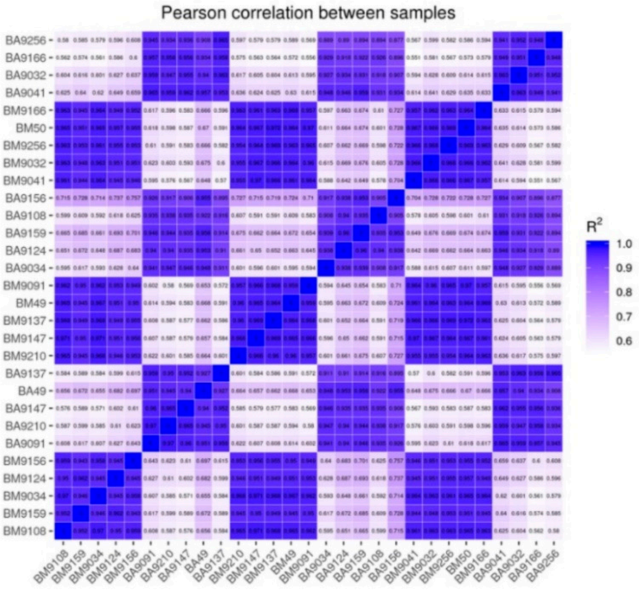

Figure S3. Volcano plots of differential gene expression analyses. **(A)** Ovine muscle differential expression comparing high-fat (Class 3) and low-fat (Class 1) carcasses. **(B)** Ovine adipose differential expression comparing high-fat (Class 3) and low-fat (Class 1) carcasses. **(C)** Bovine muscle differential expression comparing Choice and Standard USDA quality grades. **(D)** Bovine adipose differential expression comparing Choice and Standard USDA quality grades. Points represent individual genes plotted by $\log_2$ fold change and $-\log_{10}$(p-value); dashed lines indicate statistical significance thresholds as defined in the Methods.

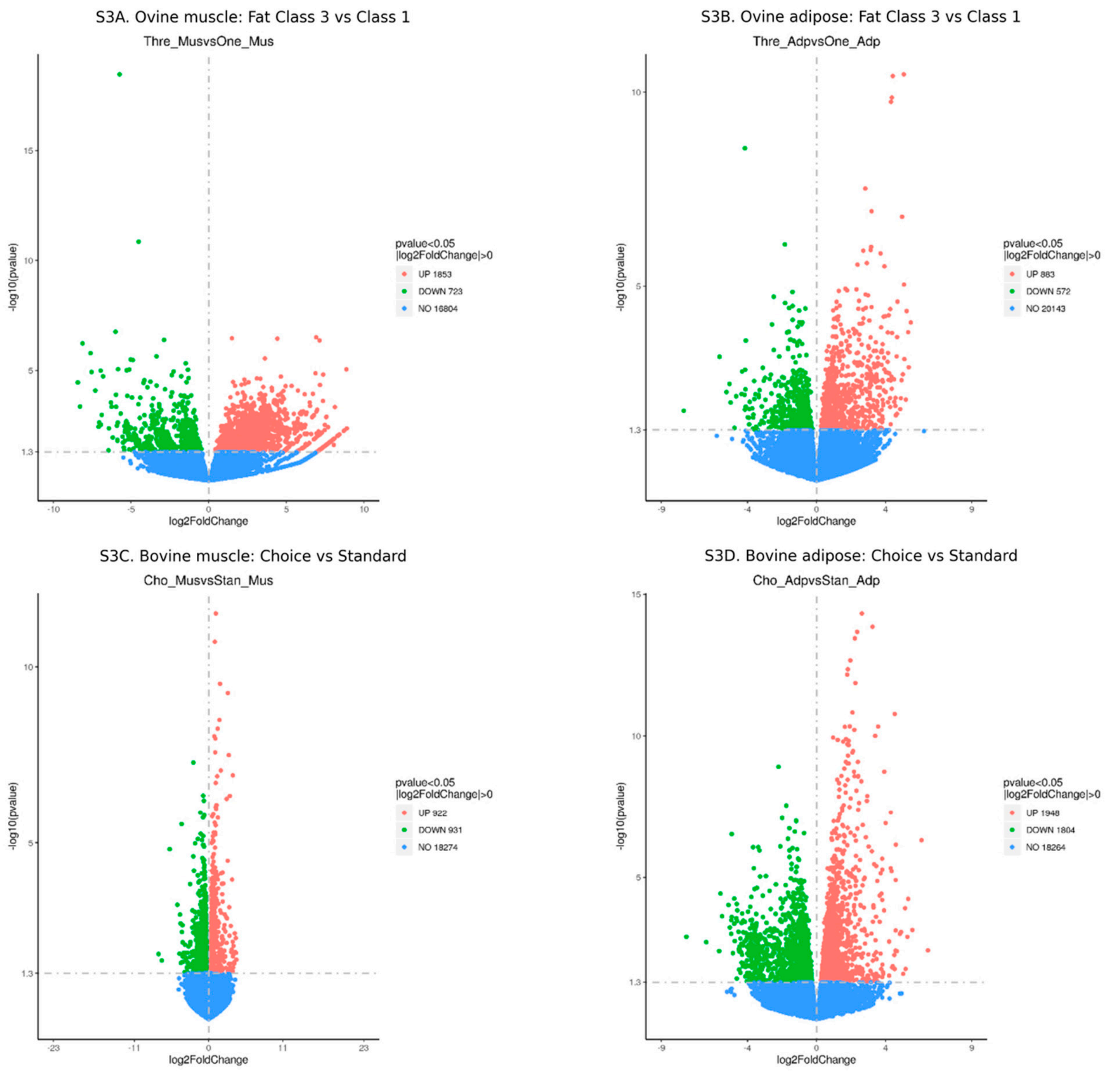

Figure S4. Pathway enrichment results—beef muscle. Enriched pathways identified by Ingenuity Pathway Analysis (IPA) from differentially expressed genes in bovine muscle between USDA quality grades (Choice vs. Standard). Pathways are ranked by significance (adjusted p-value/FDR), and the direction/extent of enrichment is summarized as shown in the plot.

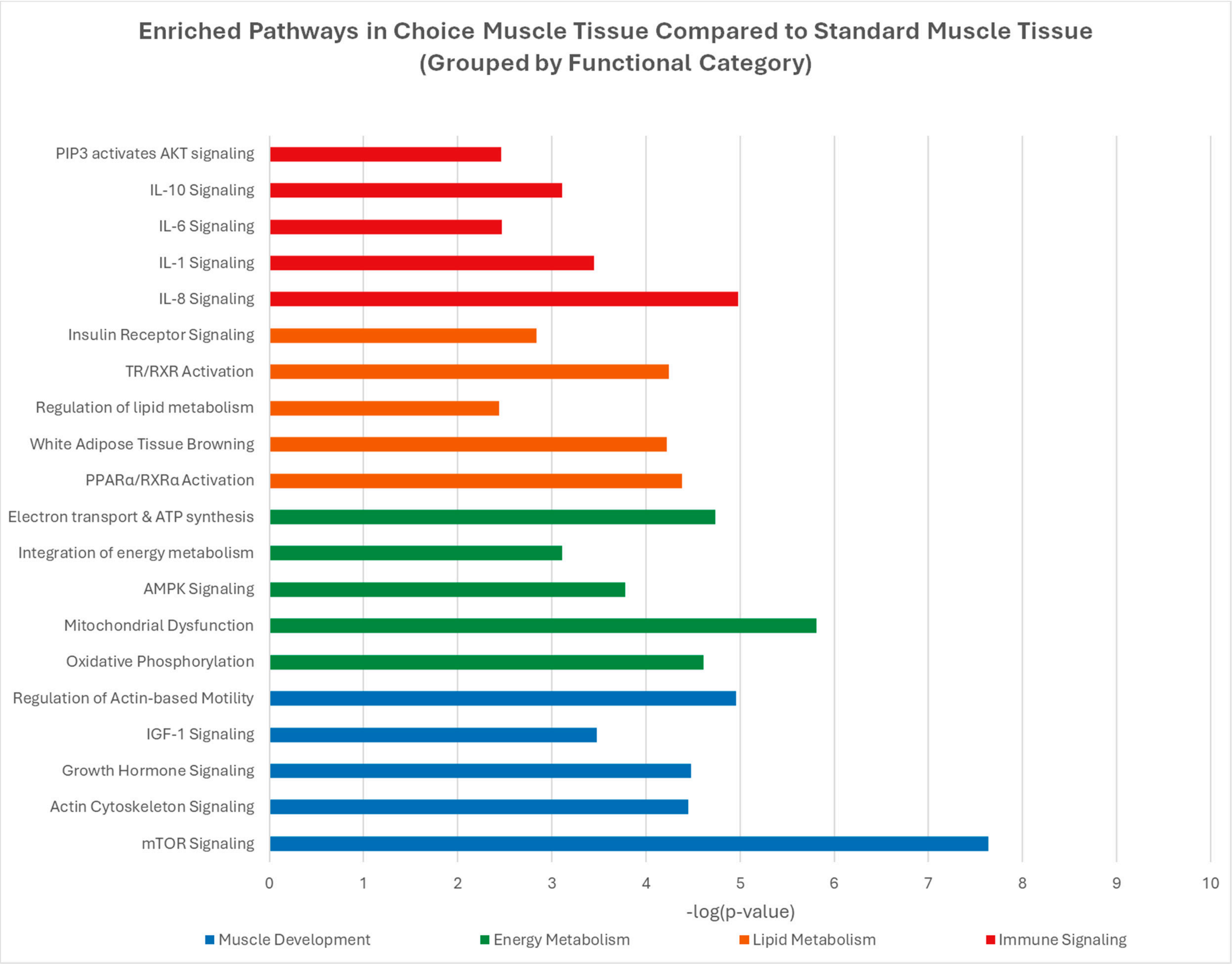
Enriched Pathways in Choice Muscle Tissue Compared to Standard Muscle Tissue
(Grouped by Functional Category)
PIP3 activates AKT signaling
IL-10 Signaling
IL-6 Signaling
IL-1 Signaling
IL-8 Signaling
Insulin Receptor Signaling
TR/RXR Activation
Regulation of lipid metabolism
White Adipose Tissue Browning
PPARα/RXRα Activation
Electron transport & ATP synthesis
Integration of energy metabolism
AMPK Signaling
Mitochondrial Dysfunction
Oxidative Phosphorylation
Regulation of Actin-based Motility
IGF-1 Signaling
Growth Hormone Signaling
Actin Cytoskeleton Signaling
mTOR Signaling
0
1
2
3
4
5
6
7
8
9
10
-log(p-value)
Muscle Development
Energy Metabolism
Lipid Metabolism
Immune Signaling

Figure S5. Pathway enrichment results—beef adipose. Enriched canonical pathways identified by Ingenuity Pathway Analysis (IPA) organized by functional category.

Stacked bar chart displaying the top enriched canonical pathways from differentially expressed genes, grouped into four functional categories: Immune/Inflammatory (red), Muscle/Cytoskeleton (blue), Lipid Metabolism (orange), and Energy/Signaling (green). Bar length represents statistical significance as −log(p-value). Neutrophil degranulation exhibited the highest significance (−log(p) = 18.0), followed by RHO GTPases Activate Formins (−log(p) = 7.26) and cholesterol biosynthesis (−log(p) = 6.44). These pathway categories reflect key biological processes associated with muscle development, adipose tissue remodeling, lipid deposition, and metabolic regulation relevant to meat quality traits. Pathways were selected based on their relevance to AMPK–mTOR energy sensing, PI3K/AKT anabolic signaling, PPAR/lipid metabolism, and immune-mediated tissue remodeling processes discussed in the main text.

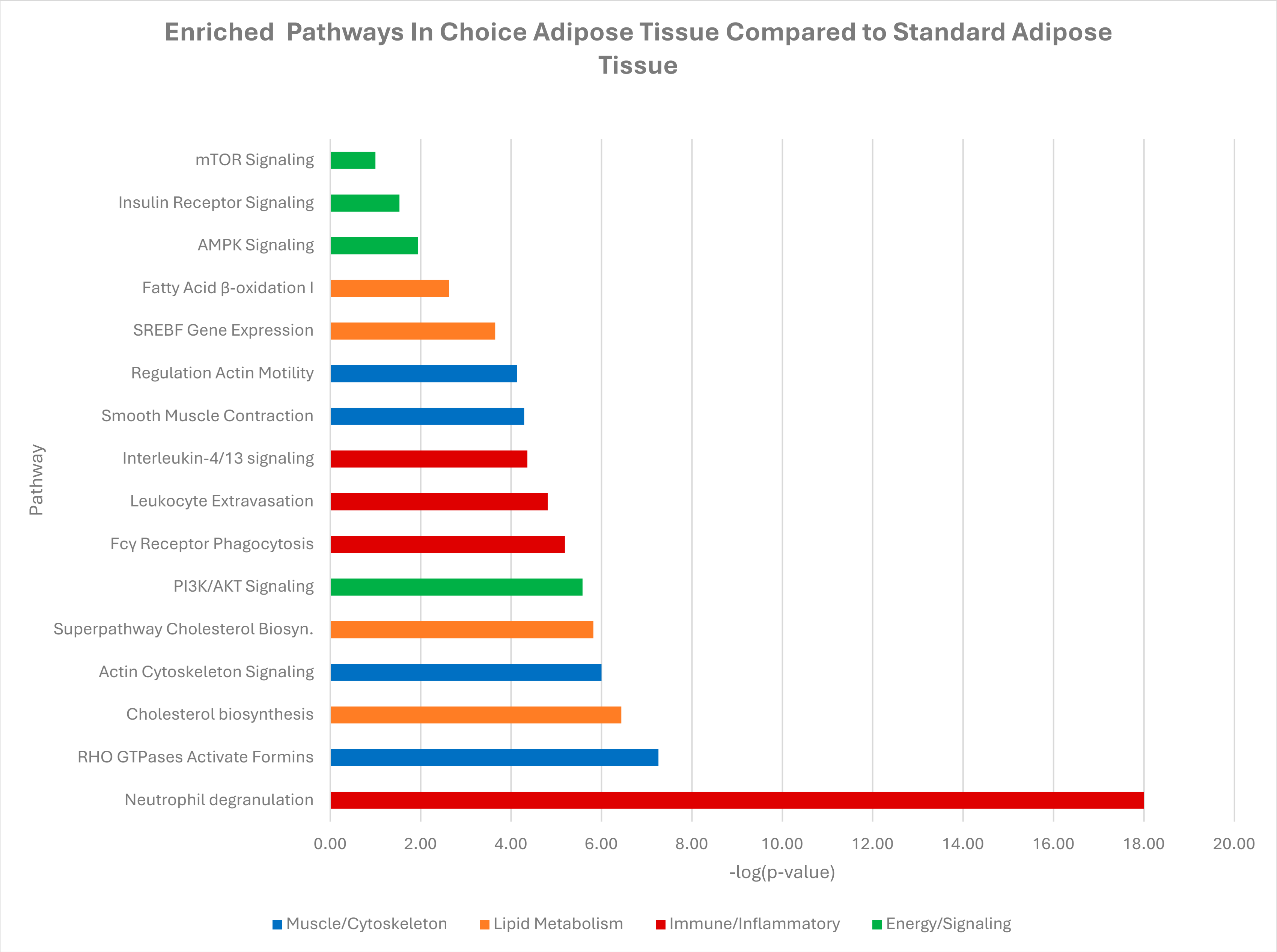
Enriched Pathways In Choice Adipose Tissue Compared to Standard Adipose Tissue
mTOR Signaling
Insulin Receptor Signaling
AMPK Signaling
Fatty Acid β-oxidation I
SREBF Gene Expression
Regulation Actin Motility
Smooth Muscle Contraction
Interleukin-4/13 signaling
Leukocyte Extravasation
Fcγ Receptor Phagocytosis
PI3K/AKT Signaling
Superpathway Cholesterol Biosyn.
Actin Cytoskeleton Signaling
Cholesterol biosynthesis
RHO GTPases Activate Formins
Neutrophil degranulation
Pathway
0.00
2.00
4.00
6.00
8.00
10.00
12.00
14.00
16.00
18.00
20.00
-log(p-value)
Muscle/Cytoskeleton
Lipid Metabolism
Immune/Inflammatory
Energy/Signaling

Figure S6. Pathway enrichment results—sheep muscle. Stacked bar chart displaying the top 20 enriched canonical pathways identified by Ingenuity Pathway Analysis (IPA) from differentially expressed genes, organized by functional category: Immune/Inflammatory (red), Muscle Development (blue), Lipid Metabolism (orange), and Energy/Glucose Metabolism (green). Bar length represents pathway significance as $-\log_{10}$(p-value). Pathways were selected based on relevance to the manuscript's central themes of muscle fiber type switching, lipid metabolism, and inflammatory signaling. The dashed line at $-\log_{10}$(p-value) = 1.3 indicates the significance threshold ($p < 0.05$).

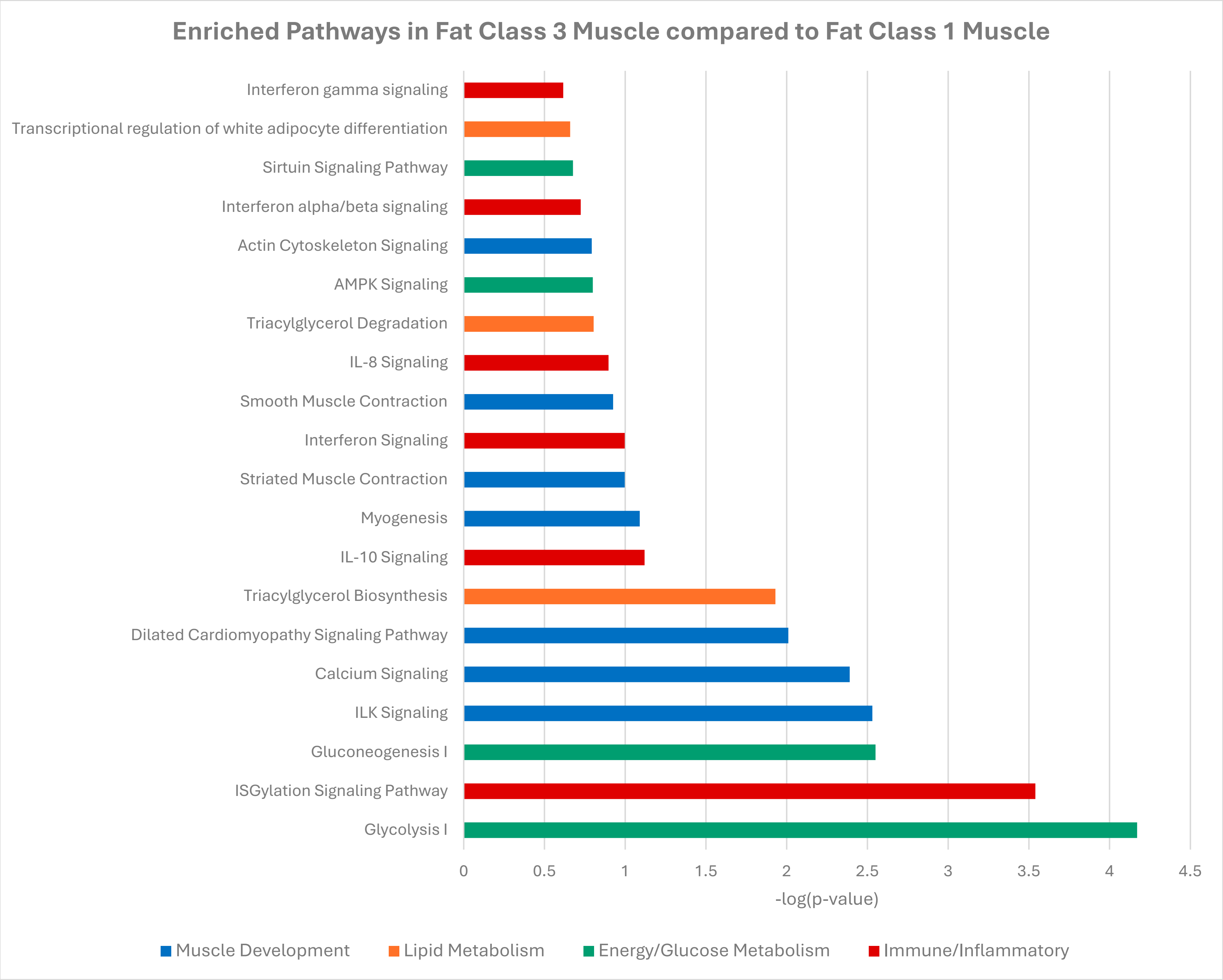

Enriched Pathways in Fat Class 3 Muscle compared to Fat Class 1 Muscle
Interferon gamma signaling
Transcriptional regulation of white adipocyte differentiation
Sirtuin Signaling Pathway
Interferon alpha/beta signaling
Actin Cytoskeleton Signaling
AMPK Signaling
Triacylglycerol Degradation
IL-8 Signaling
Smooth Muscle Contraction
Interferon Signaling
Striated Muscle Contraction
Myogenesis
IL-10 Signaling
Triacylglycerol Biosynthesis
Dilated Cardiomyopathy Signaling Pathway
Calcium Signaling
ILK Signaling
Gluconeogenesis I
ISGylation Signaling Pathway
Glycolysis I
0
0.5
1
1.5
2
2.5
3
3.5
4
4.5
-log(p-value)
Muscle Development
Lipid Metabolism
Energy/Glucose Metabolism
Immune/Inflammatory

Figure S7. Pathway enrichment results—sheep adipose. Stacked bar chart showing the most significantly enriched canonical pathways identified from differentially expressed genes. Pathways are organized into four functional categories: Immune/Inflammatory (red; including Agranulocyte Adhesion and Diapedesis, Phagosome Formation, and Pathogen Induced Cytokine Storm Signaling), Muscle Development (blue; including Striated Muscle Contraction, ILK Signaling, and cardiac conduction), Lipid/Fat Metabolism (orange; including Dilated Cardiomyopathy Signaling and Hepatic Fibrosis Signaling), and Energy Metabolism (green; including Calcium Signaling, Cardiac Hypertrophy Signaling, and Protein Kinase A Signaling). In addition, insulin/PI3K-Akt signaling pathways are highlighted in purple (including PI3K/AKT, PTEN, and IGF-1 Signaling). Bar length represents the $-\log_{10}$(p-value) from Fisher's exact test, with longer bars indicating greater statistical significance. Pathway enrichment analysis was performed using IPA (QIAGEN).

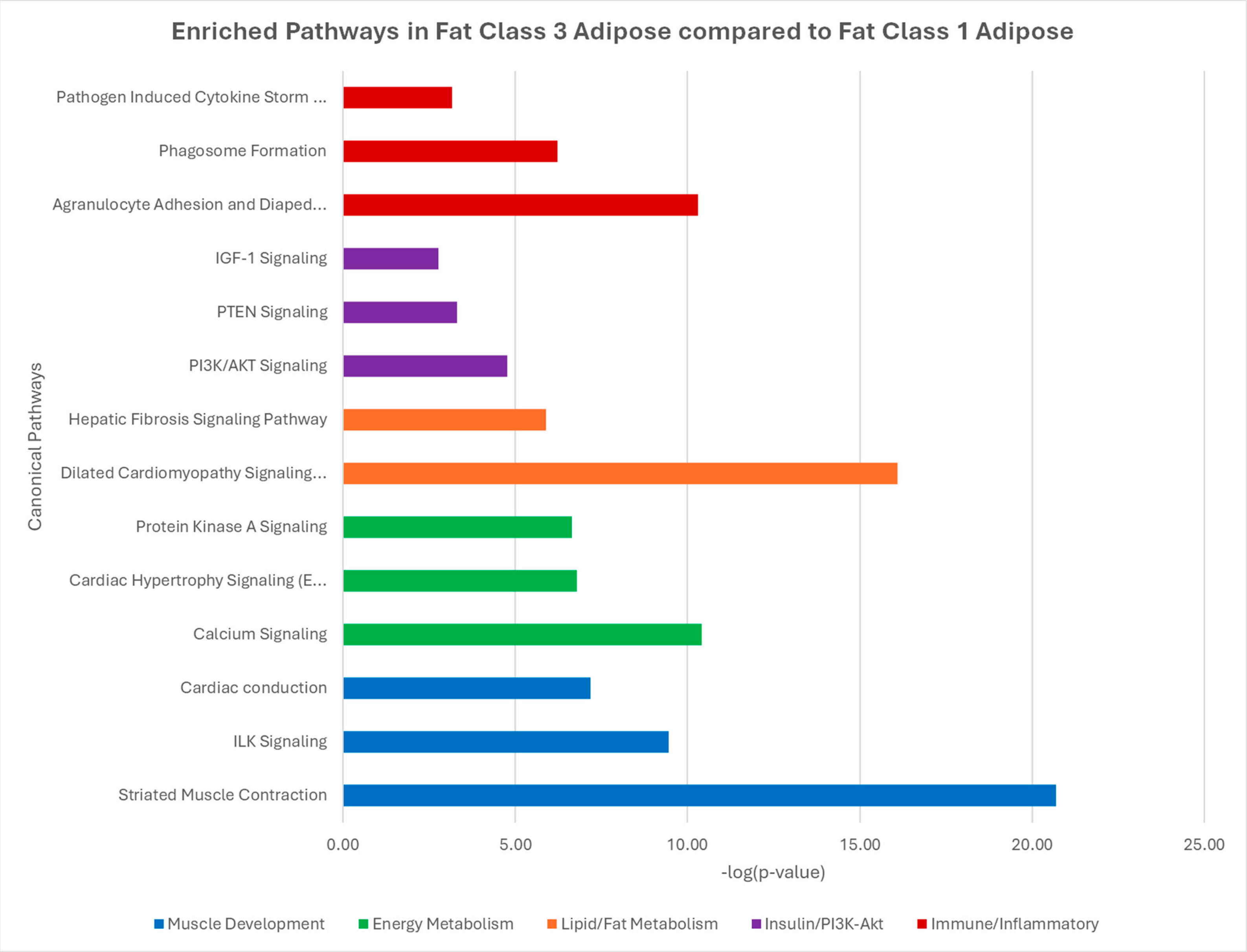
Enriched Pathways in Fat Class 3 Adipose compared to Fat Class 1 Adipose
Pathogen Induced Cytokine Storm ...
Phagosome Formation
Agranulocyte Adhesion and Diaped...
IGF-1 Signaling
PTEN Signaling
PI3K/AKT Signaling
Hepatic Fibrosis Signaling Pathway
Dilated Cardiomyopathy Signaling...
Protein Kinase A Signaling
Cardiac Hypertrophy Signaling (E...
Calcium Signaling
Cardiac conduction
ILK Signaling
Striated Muscle Contraction
Canonical Pathways
0.00
5.00
10.00
15.00
20.00
25.00
-log(p-value)
Muscle Development
Energy Metabolism
Lipid/Fat Metabolism
Insulin/PI3K-Akt
Immune/Inflammatory
